# Supplementary material for: Hematology and clinical biochemistry reference intervals for companion pigs using the ADVIA 2120 and Cobas c501
Source: PeerJ. 2025 Feb 12;13:e18968. doi: 10.7717/peerj.18968 (PMC11829631; doi:10.7717/peerj.18968)
Supplement: Supplemental Information 1 — All reagents are those provided by the manufacturer of the analyzer unless otherwise noted. [file peerj-13-18968-s001.pdf]

Supplemental Table 1:
Summary of analytic methods for CBC and biochemistry values, as provided by the manufacturers for automated values, or in accordance with UTVMC clinical pathology standard operating procedures for manual methods (nRBCs and manual WBC differential). All reagents are those provided by the manufacturer of the analyzer unless otherwise noted.

Value	Analytical Method
**RBC**	Isovolumetric sphering followed by low-angle and high-angle light scatter using the Mie theory of light scattering, with proprietary calibration factors
**HGB**	Cyanmethemoglobin method
**HCT**	Calculation, (RBCxMCV)/10
**MCV**	Mean of RBC volume histogram
**MCH**	Calculation, (HGB/RBC)x10
**MCHC**	Calculation, (HGB/[RBCxMCV])x1000
**RDW**	Calculation, 100x(standard deviation of RBC volume histogram/MCV)
**Retic**	Oxazine 750 dye for RNA content
**nRBCs**	Calculation, WBC x %nRBCs<br>%nRBCs = The percentage of nucleated cells that are nRBCs, as determined by microscopic evaluation of a blood smear.
**Platelets**	Isovolumetric sphering followed by low-angle and high-angle light scatter using the Mie theory of light scattering, with proprietary calibration factors
**Plateletcrit**	Calculation, (PLTxMPV)/10,000
**MPV**	Mean of platelet volume histogram
**WBC**	Baso channel method utilizing a reagent containing phthalic acid and a surfactant to lyse red cells and platelets, together with light scatter. If nRBCs are detected, their value is subtracted from the Baso channel WBC count and a corrected WBC count is reported.
**Neutr auto**	Light scatter versus peroxidase activity; proprietary cell gating and cluster analysis
**Lymph auto**	Light scatter versus peroxidase activity; proprietary cell gating and cluster analysis
**Mono auto**	Light scatter versus peroxidase activity; proprietary cell gating and cluster analysis
**Eos auto**	Light scatter versus peroxidase activity; proprietary cell gating and cluster analysis
**Baso auto**	Light scatter versus peroxidase activity; proprietary cell gating and cluster analysis
**LUC auto**	Light scatter versus peroxidase activity; proprietary cell gating and cluster analysis
**Neutr manual**	Calculation, WBC x %Neutr<br>%Neutr = The percentage of nucleated cells that are segmented neutrophils, as determined by microscopic evaluation of a blood smear.
**Band manual**	Calculation, WBC x %Band<br>%Band = The percentage of nucleated cells that are band neutrophils, as determined by microscopic evaluation of a blood smear.
**Lymph manual**	Calculation, WBC x %Lymph<br>%Lymph = The percentage of nucleated cells that are lymphocytes, as determined by microscopic evaluation of a blood smear.
**Mono manual**	Calculation, WBC x %Mono

	%Mono = The percentage of nucleated cells that are monocytes, as determined by microscopic evaluation of a blood smear.
**Eos manual**	Calculation, WBC x %Eos<br>%Eos = The percentage of nucleated cells that are eosinophils, as determined by microscopic evaluation of a blood smear.
**Baso manual**	Calculation, WBC x %Baso<br>%Baso = The percentage of nucleated cells that are basophils, as determined by microscopic evaluation of a blood smear.
**Tot Prot**	Colorimetric biuret method
**Albumin**	Colorimetric bromcresol green dye binding method
**Globulins**	Calculation, Total protein - Albumin
**A:G ratio**	Calculation, Albumin/Globulin
**Glucose**	Two-step enzymatic method utilizing hexokinase, with NADPH formation rate endpoint
**AST**	Two-step reaction initiated by AST catalyzing amino group transfer between L-aspartate and 2-oxoglutarate, with NADH oxidation rate endpoint
**SDH**	SDH catalyzes redox reaction between sorbitol and fructose, with NADH oxidation rate endpoint (Sekure Chemistry, Sekisui Diagnostics, LLC)
**GGT**	Enzymatic colorimetric assay in which GGT transfers a gamma-glutamyl group from a substrate to create a product measured colorimetrically
**Bilirubin**	Diazonium ion coupling
**CK**	Three-step reaction initiated by CK catalytic activity on creatine phosphate and ADP, with NADPH formation rate endpoint
**Urea**	Two-step reaction initiated by urease, with NADH oxidation rate endpoint
**Creatinine**	Kinetic colorimetry assay based on the Jaffé method
**Phosphorus**	Photometric ammonium molybdate reation
**Calcium**	Colorimetric o-Cresolphthalein complexone reaction
**Magn**	Colorimetric chlorophosphonazo III reaction
**Iron**	Colorimetric assay in acidic condition with ascorbate and FerroZine
**Chol**	Cholesterol esterase enzymatic reaction with hydrogen peroxide-mediated colorimetric detection
**Trig**	Multistep enzymatic colorimetric test initiated by lipoprotein lipase with peroxidase endpoint
**Sodium**	Ion-elective electrode
**Potassium**	Ion-selective electrode
**Chloride**	Ion-selective electrode
**TCO2**	Two-step reaction initiated by bicarbonate reacting with phosphoenolpyruvate, with an endpoint of NADH consumption
**Anion Gap**	Calculation, (Na + K) – (Cl + TCO2)
