# Supplementary material for: Hematology and clinical biochemistry reference intervals for companion pigs using the ADVIA 2120 and Cobas c501
Source: PeerJ. 2025 Feb 12;13:e18968. doi: 10.7717/peerj.18968 (PMC11829631; doi:10.7717/peerj.18968)
Supplement: Supplemental Information 2 — Abbreviations: WCI, width of the confidence interval; WRI, width of the reference interval. [file peerj-13-18968-s002.pdf]

Supplemental Table 2:
Values in which the ratio of confidence interval width to reference interval width exceeded the ideal of 0.2 for either the upper or lower reference limit. Abbreviations: WCI, width of the confidence interval; WRI, width of the reference interval.

**Hematology, WCI/WRI>0.2 for *Lower* Reference Limit**	
**Measurand**	**WCI/WRI**
HGB	0.27
MCHC	0.50
RBC	0.24
**Hematology, WCI/WRI>0.2 for *Upper* Reference Limit**	
**Measurand**	**WCI/WRI**
Band, Manual	0.57
Baso, Auto	0.77
Baso, Manual	1.02
Eos, Auto	0.88
Eos, Manual	1.03
Fibrinogen, Heat Precipitation	0.45
Hematocrit	0.34
Hemoglobin	0.54
Lymph, Auto	0.22
Lymph, Man	0.26
Mono, Auto	0.42
Mono, Man	0.46
MCH	0.38
MCHC	0.27
MCV	0.44
MPV	0.28
Neutr, Auto	0.48
Neutr, Man	0.45
nRBC	0.71
Platelet	0.22
Plateletcrit	0.79
RBC	0.23
RDW	1.24
Total protein, Refractometer	0.21
WBC count	0.35

**Clinical Chemistry, WCI/WRI>0.2 for *Lower* Reference Limit**	
**Measurand**	**WCI/WRI**
Albumin	0.25
Globulin	0.21
Phosphorus	0.21
TCO2	0.36
**Clinical Chemistry, WCI/WRI>0.2 for *Upper* Reference Limit**	
**Measurand**	**WCI/WRI**
Albumin to Globulin Ratio	1.37
Anion Gap	0.68
AST	0.73
Bilirubin	0.52
Cholesterol	0.29
Creatine Kinase	0.70
Creatinine	0.36
GGT	1.14
Glucose	0.38
Iron	0.52
Magnesium	0.57
Phosphorus	1.28
Potassium	0.36
SDH	1.34
Triglycerides	0.48
Urea	0.26
