# Supplementary material for: Hematology and clinical biochemistry reference intervals for companion pigs using the ADVIA 2120 and Cobas c501
Source: PeerJ. 2025 Feb 12;13:e18968. doi: 10.7717/peerj.18968 (PMC11829631; doi:10.7717/peerj.18968)
Supplement: Supplemental Information 3 [file peerj-13-18968-s003.pdf]

Supplemental Table 3:

Porcine Reference Intervals from 7 publications, Comparison of Methods.

First Author	Schaefer	Dimistrakakis	Li	Perri	Verheyen	Brockus	Radin
**Year**	2025	2022	2021	2017	2007	2005	1986
**Housing**	Client-owned companion pigs	Single herd, research pigs	Specific pathogen free research pigs	20 production swine farms	8 different breeding herds on different farms	Single rescue farm	Research colony breeding stock
**Breed**	Varied, mostly potbellied, mini pigs, and mixed breed	Yorkshire	Landrace	Not reported	Landrace, Large white, and hybrid	Vietnamese potbellied pigs	Yucatan miniature swine
**Age**	5 months to 11 years	3-4 months	1 month	18-26 days	Not reported	2-10 years	8-54 months
**Reproductive Status**	Sexually mature females (spayed and intact) and males (castrated and intact)	Juvenile females	Juvenile females and males	Juvenile females and males	Pregnant and lactating sows	Sexually mature females and males	Sexually mature intact females and males
**Number of individuals**	94	124	105	1032 for hematology, 200 for chemistry	132	100	30
**Hematology Instrument**	ADVIA 2120	Abaxis VetScan HM5	BC-5000 Vet Hematology Analyzer	ADVIA 2120	Not evaluated	Baker 9000	Coulter
**Chemistry Instrument**	Cobas c501	Abaxis VetScan VS2	D240V Clinical Chemistry Analyzer	Cobas c501	Beckman Synchron CX 4	Abbott Spectrum Series II	Rotochem
**Statistical method**	Compliant with ASVCP guidelines	Compliant with ASVCP guidelines	Compliant with ASVCP guidelines	Compliant with ASVCP guidelines	Central 95%	Mean +/- 2 standard deviations	Mean +/- 2 standard deviations
