# Supplementary material for: Hematology and clinical biochemistry reference intervals for companion pigs using the ADVIA 2120 and Cobas c501
Source: PeerJ. 2025 Feb 12;13:e18968. doi: 10.7717/peerj.18968 (PMC11829631; doi:10.7717/peerj.18968)
Supplement: Supplemental Information 4 — Unit conversions were applied to those values published in SI units. Abbreviation: NR = not reported. Note: Verheyen 2007 published only clinical chemistry values so is not included in this table. [file peerj-13-18968-s004.pdf]

Supplemental Table 4:

Porcine Reference Intervals from 6 publications, Hematology and Refractometric Values. Unit conversions were applied to those values published in SI units. Abbreviation: NR = not reported. Note: Verheyen 2007 published only clinical chemistry values so is not included in this table.

First Author	Schaefer	Dimistrakakis	Li	Perri	Brockus	Radin
**RBC** (10^{12}/L)	**2.70 – 7.67**	4.80 – 7.11	4.29 – 7.65	4.8 – 7.3	3.6 – 7.8	5.4 – 8.6
**HGB** (g/dl)	**7.8 – 16.0**	8.2 – 11.7	9.9 – 14.8	9.3 – 13.6	7.8 – 16.2	12.5 – 17.3
**HCT** (%)	**22.2 – 43.7**	25.4 – 38.8	22.3 – 46.2	30 – 50	22 – 50 (PCV)	36.4 – 52.8
**MCV** (fL)	**52.0 – 78.9**	NR	41.6 – 68.7	53.0 – 79.0	55 – 71	57.0 – 71.8
**MCH** (pg)	**18.4 – 26.9**	NR	12.9 – 22.8	15.0 – 23.0	18 – 24	18.8 – 24.0
**MCHC** (g/dl)	**32.1 – 35.9**	NR	27.4 – 33.0	27.5 – 31.7	31 – 36	31.6 – 34.8
**RDW** (%)	**13.5 – 23.4**	NR	NR	14.3 – 26.0	NR	NR
**Retic** (10^9/L)	**18.7 – 131.4***	NR	NR	NR	NR	NR
**nRBCs** (10^9/L)	**0 – 0.2**	NR	NR	NR	NR	NR
**Platelets** (10^9/L)	**145 – 504**	162 – 449	118 – 660	172 - 833	204 – 518	201 – 680
**Plateletcrit** (%)	**0.20 – 0.47**	NR	0.11 – 0.65	NR	NR	NR
**MPV** (fL)	**6.9 – 16.7**	NR	7.5 – 11.9	7.5 – 11.9	8.4 – 12.4	NR
**WBC** (10^9/L)	**4.3 – 19.5**	9.9 – 22.0	6.0 – 23.6	6.0 – 21.7	5.2 – 17.9	6.6 – 18.6
**Neutr auto** (10^9/L)	**1.1 – 13.3**	2.0 – 8.8	1.6 – 14.0	NR	NR	NR
**Lymph auto** (10^9/L)	**1.9 – 6.3**	6.4 – 14.4	2.3 – 14.9	NR	NR	NR
**Mono auto** (10^9/L)	**0.1 – 0.9**	0.1 – 0.4	0.1 – 0.9	NR	NR	NR
**Eos Auto** (10^9/L)	**0 – 1.0**	NR	0 – 0.5	NR	NR	NR
**Baso auto** (10^9/L)	**0 – 0.1**	NR	0 – 0.2	NR	NR	NR
**LUC auto** (10^9/L)	**0 – 0.4**	NR	NR	NR	NR	NR
**Neutr manual** (10^9/L)	**1.0 – 14.6**	NR	NR	NR	0 – 11.4	NR
**Band manual** (10^9/L)	**0 – 0.2**	NR	NR	NR	0 – 0.2	NR
**Lymph manual** (10^9/L)	**1.5 – 6.6**	NR	NR	NR	0.8 – 9.8	NR
**Mono manual** (10^9/L)	**0 – 1.4**	NR	NR	NR	0 – 0.7	NR
**Eos manual** (10^9/L)	**0 – 1.7**	NR	NR	NR	0 – 0.7	NR
**Baso manual** (10^9/L)	**0 – 0.5**	NR	NR	NR	0 – 0.6	NR
**Total protein, refractometry** (g/dl)	**5.7 – 8.7**	NR	NR	NR	NR	NR
**Fibrinogen heat prec** (mg/dl)	**0 – 600**	NR	NR	NR	100 – 400	NR

*Data presented for reticulocytes is the range (minimum to maximum), not a reference interval, due to the small sample size (n=20).
