# Supplementary material for: Hematology and clinical biochemistry reference intervals for companion pigs using the ADVIA 2120 and Cobas c501
Source: PeerJ. 2025 Feb 12;13:e18968. doi: 10.7717/peerj.18968 (PMC11829631; doi:10.7717/peerj.18968)
Supplement: Supplemental Information 5 — Unit conversions applied to values published in SI units. Abbreviation: NR = not reported. Verheyen 2007 included results from each sow at 92-96 days gestation (A), 106-110 days gestation (B), 5-9 days lactation (C), and 19-23 days lactation. [file peerj-13-18968-s005.pdf]

Supplemental Table 5:

Porcine Reference Intervals from 7 publications, Clinical Chemistry. Unit conversions applied to values published in SI units. Abbreviation: NR = not reported. Verheyen 2007 included results from each sow at 92-96 days gestation (A), 106-110 days gestation (B), 5-9 days lactation (C), and 19-23 days lactation.

First Author	Schaefer	Dimistrakakis	Li	Perri	Verheyen	Brockus	Radin
**Tot Prot** (g/dl)	**5.4 – 8.2**	5.1 – 7.2	NR	4.1 – 5.5	(A) 6.5 – 9.0<br>(B) 6.2 – 8.7<br>(C) 6.2 – 9.5<br>(D) 6.6 – 9.5	6.6 – 8.9	6.1 – 8.9
**Albumin** (g/dl)	**3.1 – 5.3**	2.5 – 4.4	2.4-4.8	2.5 – 4.6	(A) 3.3 – 4.8<br>(B) 3.4 – 4.9<br>(C) 3.5 – 4.9<br>(D) 3.5 – 4.7	3.6 – 5.0	3.9 – 5.5
**Globulins** (g/dl)	**1.0 – 3.9**	1.8 – 4.1	NR	0.5 – 2.5	(A) 2.6 – 4.7<br>(B) 2.3 – 4.5<br>(C) 2.2 – 5.2<br>(D) 2.5 – 5.6	NR	1.6 – 4.0
**A:G ratio** (g/dl)	**0.9 – 4.2**	NR	NR	1.2 – 8.4	NR	NR	0.8 – 2.8
**Glucose** (mg/dl)	**35 – 197**	52 – 154	38 – 105	90 – 145	NR	60 – 175	36 – 123
**AST** (U/L)	**13 – 94**	NR	19 – 105	18 – 84	(A) 13 – 95<br>(B) 13 – 81<br>(C) 19 – 198<br>(D) 22 – 173	16 – 64	10 – 56
**SDH** (U/L)	**0 – 8.0**	NR	NR	NR	NR	NR	NR
**GGT** (U/L)	**34 – 141**	NR	NR	14 – 64	(A) 7 – 63<br>(B) 7 – 120<br>(C) 7 – 177<br>(D) 8 – 137	15 – 56	NR
**Bilirubin** (mg/dl)	**0 – 0.4**	0.2 – 0.5	0.2 – 0.9	0.1 – 1.1	(A) 0.2 – 1.2<br>(B) 0.2 – 1.1<br>(C) 0.1 – 0.7<br>(D) 0.2 – 1.0	0.2 – 0.5	0 – 0.3
**CK** (U/L)	**209 – 3850**	NR	NR	146 – 870	NR	213 – 2852	48 – 288
**Urea** (mg/dl)	**2 – 18**	2 – 11	3 – 19	3 – 14	(A) 6 – 16<br>(B) 6 – 14<br>(C) 6 – 20<br>(D) 6 – 19	4 – 15	9 – 29
**Creatinine** (mg/dl)	**0.6 – 2.3**	0.9 – 1.9	0.7 – 1.6	0.6 – 1.4	(A) 1.8 – 3.7<br>(B) 1.7 – 3.4<br>(C) 1.4 – 2.7<br>(D) 1.4 – 2.5	1.0 – 2.3	1.2 – 2.0
**Phos** (mg/dl)	**4.0 – 8.0**	6.8 – 10.6	4.1 – 12.6	8.1 – 11.8	(A) 4.3 – 10.2<br>(B) 4.6 – 9.9<br>(C) 2.8 – 12.4<br>(D) 2.8 – 8.1	NR	5.1 – 8.1
**Calcium** (mg/dl)	**9.1 – 10.9**	9.2 – 11.2	8.1 – 15.2	9.6 – 12.4	NR	NR	9.6 – 11.6
**Magn** (mmol/l)	**0.7 – 1.0**	NR	NR	0.9 – 1.5	NR	NR	NR
**Iron** (µg/dl)	**51.5 – 208.7**	NR	NR	22.0 – 251.0	NR	NR	NR
**Chol** (mg/dl)	**28 – 153**	NR	43 – 141	85 – 325	NR	NR	38 – 165
**Trig** (mg/dl)	**8.4 – 114.5**	NR	9 – 82	NR	NR	NR	NR
**Sodium** (mmol/l)	**135 – 146**	129 – 143	NR	122 – 145	NR	139 – 149	144 – 153
**Potassium** (mmol/l)	**3.4 – 4.9**	3.5 – 4.7	NR	3.9 – 6.2	NR	3.7 – 5.0	4.0 – 5.2
**Chloride** (mmol/l)	**96 – 107**	NR	83 – 113	87 – 103	NR	106 – 113	94 – 114
**TCO2** (mmol/l)	**19.1 – 34.2**	NR	NR	19.0 – 31.0	NR	8.0 – 31.0	NR
**Anion Gap** (mmol/l)	**10 – 36**	NR	NR	16.0 – 28.3	NR	8 – 36	NR
